# Analysis of Acoustic Absorption Coefficients and Characterization of Epoxy Adhesive Compositions Based on the Reaction Product of Bisphenol A with Epichlorohydrin Modified with Fillers

**DOI:** 10.3390/ma17184452

**Published:** 2024-09-10

**Authors:** Izabela Miturska-Barańska, Anna Rudawska, Lydia Sobotova, Miroslav Badida, Ewa Olewnik-Kruszkowska, Miroslav Müller, Monika Hromasová

**Affiliations:** 1 Department of Production Computerisation and Robotisation, Faculty of Mechanical Engineering, Lublin University of Technology, Nadbystrzycka 36, 20-618 Lublin, Poland; a.rudawska@pollub.pl; 2 Department of Business Management and Environmental Engineering, Faculty of Mechanical Engineering, Technical University of Kosice, Letná 9, 042 00 Košice, Slovakia; lydia.sobotova@tuke.sk (L.S.); miroslav.badida@tuke.sk (M.B.); 3 Department of Physical Chemistry and Physicochemistry of Polymers, Faculty of Chemistry, Nicolaus Copernicus University in Toruń, Gagarina 7, 87-100 Toruń, Poland; olewnik@umk.pl; 4 Department of Material Science and Manufacturing Technology, Faculty of Engineering, Czech University of Life Sciences, Kamýcká 129, 165 21 Prague, Czech Republic; muller@tf.czu.cz; 5 Department of Electrical Engineering and Automation, Faculty of Engineering, Czech University of Life Sciences, Kamýcká 129, 165 21 Prague, Czech Republic; hromasova@tf.czu.cz

**Keywords:** adhesive modification, epoxy composition, sound absorption test, SEM analysis

## Abstract

Material development in acoustic engineering plays a significant role in various applications, such as industrial noise control. It is important and relevant to consider alternative materials capable of reducing noise levels in different frequency ranges. One commonly used material in engineering structures is epoxy adhesive compositions. Favoring the use of adhesive compositions are their main characteristics, including weight reduction in structures, corrosion resistance, relatively low manufacturing costs, and high mechanical strength. This paper aims to discuss the relationship between the mechanical properties of modified epoxy adhesives, their structure, and sound absorption efficiency. The subjects of this study were specimens of an epoxy composition in the cured state. Acoustic absorption coefficients were evaluated using a dual-microphone impedance tube, and tensile, compressive, and bending strength properties were determined using a testing machine. The impact strength of the compositions was also investigated. An analysis of the structure of the adhesives in the cured state was carried out using a scanning electron microscope. The test specimens were made from Epidian 5 epoxy resin cured with a polyamide PAC curing agent. Nanobent ZR2 aluminosilicate in an amount of 1%, CaCO_3_ calcium carbonate in an amount of 5%, and CWZ-22 activated carbon in an amount of 20% were used as modifiers. The conducted studies revealed that the highest tensile strength was obtained for the adhesive composition with the addition of ZR2 filler. The highest compressive strength was exhibited by the adhesive composition with the addition of CWZ-22 filler. The highest flexural strength was demonstrated by the unmodified composition. For all the tested adhesive compositions, low sound absorption values were achieved, with a maximum of approximately 0.18. From the perspective of the reduction index *R*, it was observed that these samples performed better in reduction than in absorption. The best values were achieved in the compositions modified with CaCO_3_.

## 1. Introduction

Sound-absorbing materials are increasingly utilized to mitigate the adverse effects of hard and rigid internal surfaces and to diminish resonant noise. The advancement of materials in acoustic engineering holds significant importance across various applications, including room acoustics control, industrial noise control, and automotive noise control [1,2]. It is crucial and pragmatic to explore alternative materials capable of reducing noise levels across different frequency ranges.

One material commonly utilized in engineering structures is epoxy adhesive compositions. The key advantages favoring the use of adhesive compositions include structural weight reduction, corrosion resistance, relatively low manufacturing costs, vibration-damping capability, and high mechanical strength. Further benefits of adhesive compositions that promote their usage encompass weather and wear resistance, high fatigue strength, and thermal stability. Epoxy adhesives have served as the primary family of bonding agents for structural bonding of metals and composite materials for many years [3,4]. The versatility of epoxy adhesives arises from the numerous combinations of epoxy resins and curing agents, which provide diverse chemical compositions and curing methods, resulting in various molecular structures in the resulting polymer [5,6]. The term ‘epoxy adhesives’ frequently describes compositions based on various epoxy resins, which are among the most commonly used matrices for obtaining adhesive compositions used as structural adhesives. The most prevalent is DGEBA—bisphenol A diglycidyl ether, also known as dianium diglycidyl ether—a resin with an average molecular weight of 180–200 g/mol [6,7,8].

While bonding technology has a long history, its development over centuries continues to progress with vigor. The quest for new solutions persists, driven by the widespread use of bonding technology and epoxy compositions. These compositions not only serve as bonding agents but can also function as construction materials in their cured state. This shift is primarily propelled by growing environmental awareness, evolving laws, and regulations that demand sustainable and ecologically efficient technical applications. Consequently, there is an increasing adoption of naturally derived plastics of natural origin. Jayamani et al., in their paper [9], highlighted that materials of natural origin, particularly natural fibers used as reinforcement in thermoplastic resin matrices, offer economical, readily biodegradable, renewable, and non-toxic properties. A growing body of research, as reported in the literature [10,11,12,13,14], focuses on modifying epoxy adhesive compositions. By incorporating specific compounds, the desired properties of the adhesive composition and resulting adhesive joints can be achieved [15]. Wang et al., in their paper [16], investigated a porous fiber material for its sound absorption properties and identified flow resistivity and sound absorption coefficient as crucial material parameters. Materials used for sound absorption and attenuation are typically much softer than solid materials and possess a porosity greater than 90% [17]. The presence of pores or voids in the material structure plays a pivotal role, serving as a medium for sound wave scattering [18,19,20]. When sound waves encounter these pores, they vibrate and dissipate energy through thermal and viscous losses within the internal pores and ducts [21].

Considering the aspects outlined above, including the extensive use of epoxy adhesives and the demand for new structures with improved sound absorption capabilities, it appears reasonable in the described research to attempt to ascertain the relationship between the mechanical properties of modified epoxy adhesives, their structure, and sound absorption performance.

Recent advancements in sound-absorbing materials have focused on developing alternative materials that can effectively reduce noise levels across different frequency ranges. Traditionally, materials such as porous fibers, foams, and other soft, highly porous structures have been the go-to choices for sound absorption due to their ability to scatter sound waves through their porous structures. However, there is a growing interest in multifunctional materials that combine structural strength with sound absorption capabilities. This shift is driven by the need for materials that not only offer noise reduction but also meet other critical engineering requirements such as mechanical strength, durability, and environmental sustainability.

One of the emerging trends in this area is the use of modified epoxy adhesives as sound-absorbing materials. Epoxy adhesives, traditionally used for their strong bonding properties, are being explored for their potential in sound absorption, especially when modified with various fillers. These fillers, such as aluminosilicates, calcium carbonate, and activated carbon, can influence both the mechanical and acoustic properties of the cured epoxy, making them a versatile option for applications where both structural integrity and sound absorption are required.

The aim of this research was to conduct studies to investigate the impact of adhesive composition modifications on the properties of adhesive compositions, particularly focusing on the relationship between the mechanical properties of modified epoxy adhesives, their structure, and sound-absorbing properties. Cured adhesive samples were tested in this study. An epoxy resin named Epidian 5, cured with a polyamide PAC curing agent, served as the matrix material. The modifying agents included Nanobent ZR2 aluminosilicate at 1 wt.%, CaCO_3_ calcium carbonate at 5 wt.% and CWZ-22 activated carbon at 20 wt.%. Sound absorption coefficients were evaluated using a dual-microphone impedance tube, and tensile, compressive, and bending strength properties were determined using a testing machine. Scanning electron microscopy was used to analyze the structure of the materials.

The study described in this paper contributes significantly to this emerging field by exploring the relationship between the mechanical properties, structure, and sound absorption performance of modified epoxy adhesives. The novelty of this work lies in the specific combination of fillers used—Nanobent ZR2 aluminosilicate, CaCO_3_ calcium carbonate, and CWZ-22 activated carbon—and the systematic investigation of how these modifications impact both the tensile and compressive strengths, as well as the sound absorption coefficients.

This research addresses a gap in the literature by not only focusing on the traditional structural applications of epoxy adhesives but also exploring their potential as multifunctional materials that can meet the dual demands of mechanical strength and acoustic performance. The use of scanning electron microscopy (SEM) to analyze the structural changes induced by the fillers adds another layer of depth to this study, allowing for a better understanding of the microstructural factors that contribute to the observed properties.

Overall, this work represents a novel approach to material design, particularly in the context of developing eco-friendly and high-performance materials for engineering applications. It paves the way for further exploration of epoxy-based composites in sound absorption and other multifunctional applications.

## 2. Experimental

**Figure 1:**
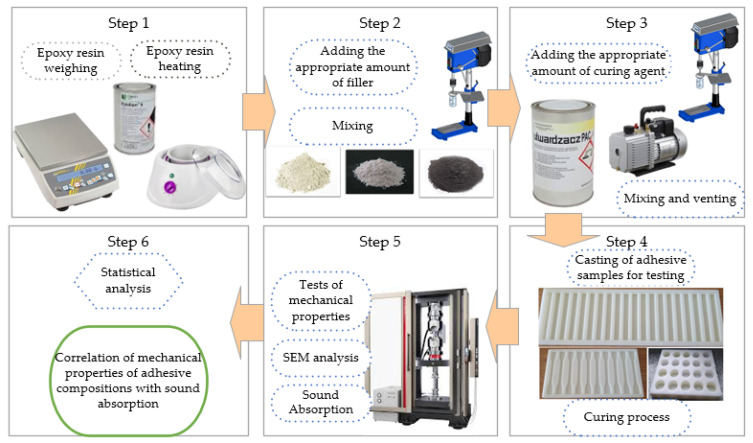
Procedure for creating and testing the samples.

The test subjects comprised samples of the epoxy composition in the cured state. The procedure for creating and testing the samples is shown in Figure 1.

For each test performed, 10 samples were prepared and tested.

### 2.1. Materials

The matrix of the composition utilized in this study was Epidian 5 epoxy resin (CIECH S.A., Sarzyna, Poland). This particular epoxy resin originates from the reaction between bisphenol A and epichlorohydrin, resulting in a pure form. Its notable features include exceptional adhesion to most plastics, resistance to chemicals and harsh environmental conditions, as well as favorable electrical properties [14,22,23]. Widely employed in diverse industries, such as the production of fiberglass laminates and bonding of metals, ceramics, and thermosetting plastics, Epidian 5 epoxy resin and its derivatives offer versatile applications. Additionally, adhesives incorporating this resin serve as protective coatings against corrosion and electrical insulation in construction projects. Table 1 provides a summary of the performance characteristics of the epoxy resin under investigation.

The polyamide PAC curing agent (CIECH S.A., Sarzyna, Poland) was used to cure the resin. The PAC curing agent comprises fatty acids, C18-unsaturated dimers, and polymeric reaction products with triethylenetetramine and is commonly used for curing liquid epoxy resins. This curing agent enhances the elasticity and impact strength of the composition, making it suitable for connections subjected to deformation. For instance, it is utilized in boat-building to join wooden or polyester–glass laminate elements, in bonding rubber with metal, thin sheets, plywood, and in casting elements for electrical engineering and electronics. The PAC curing agent belongs to the group of slow-reacting curing agents. The properties of the curing agent used in this study are outlined in Table 2.

The primary objective of incorporating fillers into adhesive compositions is to enhance specific performance properties. Additionally, considering the dynamic technological advancements, environmental considerations also play a significant role. The initial filler used was a highly granular filler, referred to as ZR2 NanoBent (Zakłady Górniczo-Metalowe “Zębiec” S.A., Zębiec, Poland), characterized by its particle size on the micro and nano scales. It is an aluminosilicate modified with a quaternary ammonium salt. Montmorillonite ZR2 serves as an additive with a dual effect: thixotropic and biocidal. The properties of the nanofiller utilized are summarized in Table 3.

ZR2 NanoBent is a bentonite that has undergone special modification. Bentonite, inherently hydrophilic, presents challenges in dispersion within most organic engineering polymers due to its natural properties. To address this, bentonites are modified using their layered (lamellar) structure. Typically, bentonites are modified with amine salts to increase the interlamellar distance (exfoliation). This modification process results in the hydrophobization of the filler, enabling polymer or monomer molecules to more easily penetrate between the layers of modified bentonite, thereby facilitating the production of composite or nanocomposite.

In this research, the second filler employed was calcium carbonate (CaCO_3_) in powdered form (Zakłady Przemysłu Wapienniczego Trzuskawica S.A., Siatkówka, Poland), possessing a molecular weight of 100.09 g/mol. Its typical concentration is approximately 98.23%, with a concentration range spanning from 92% to 99%. The fundamental physical and chemical characteristics of calcium carbonate are outlined in Table 4.

In this study, the third filler employed was CWZ-22 activated carbon in particulate form (PPH STANLAB SP. Z O.O., Lublin, Poland), with a molar mass of 12.01 g/mol. The production of activated carbons relies on natural organic raw materials characterized by a polymeric structure. Primary sources encompass wood (constituting 35% of total raw material consumption), hard coal (28%), lignite (14%), peat (10%), and, in specific locales, waste materials such as nut shells or fruit stones (10%). Introducing carbon as a powdered filler into the cured polymer matrix can notably influence both thermal and mechanical properties. From a structural perspective, mechanical properties are of primary interest. Understanding these properties, which are determined under applied forces of specific distribution and magnitude, enables the approximate prediction of the composite material’s behavior under real-world conditions. Additionally, the presence of carbon can induce a porous structure in the adhesive, as demonstrated in one of the authors’ patent applications [25]. Table 5 provides an overview of the fundamental properties of the carbon utilized in this investigation.

In this paper, abbreviated designations were used for easier identification of the tested adhesive compositions. The method of marking individual compositions is shown in Table 6.

### 2.2. Preparation of Samples

**Figure 2:**
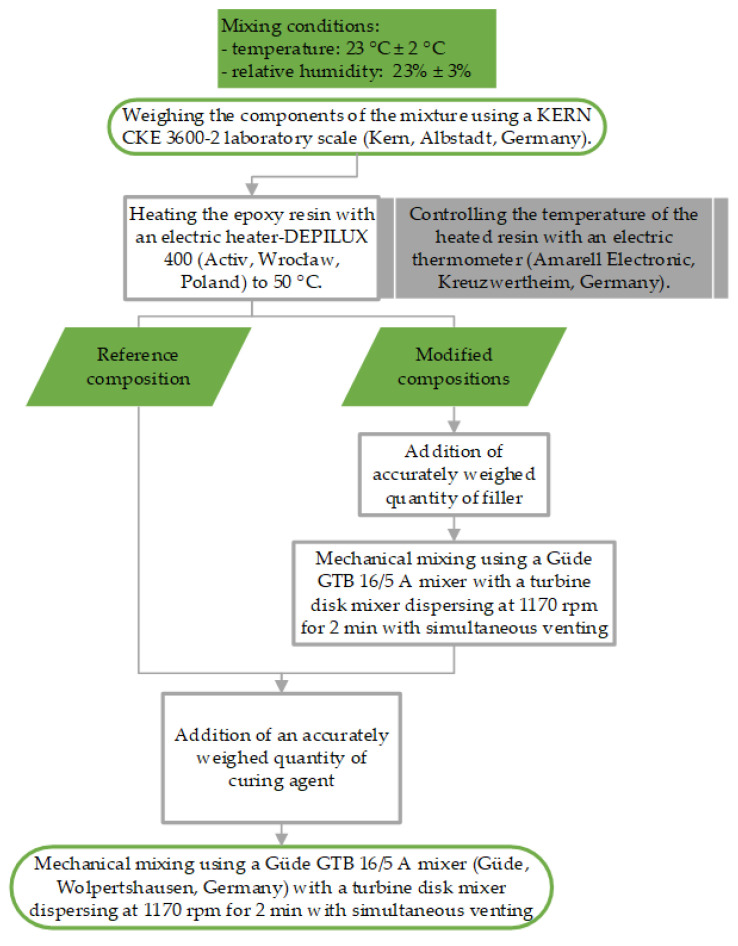
Mixing steps for adhesive compositions.

The quantities of fillers employed were chosen through our independent experimental investigations and a comprehensive review of the existing literature [26,27,28,29,30,31,32]. The stages involved in mixing the components of the adhesive compositions used in this study are shown in Figure 2.

The mixed adhesive compositions were cast using specially developed silicone molds and then cured for 7 days under conditions identical to those present during mixing. The dimensions and geometry of the samples are shown in Table 7.

### 2.3. Test Methods

#### 2.3.1. Scanning Electron Microscopy (SEM)

The scanning electron microscopy (SEM) analysis was conducted utilizing the Tescan MIRA3 microscope (Tescan Orsay Holding, Brno-Kohoutovice, Czech Republic). SEM tests were carried out at an accelerating voltage of 10.0 kV, with a working distance of 15 mm. An +BSE secondary electron detector was used to image the adhesive compositions, and the samples were sputtered with gold using Quarum Q150R ES (Tescan Brno s.r.o., Brno, Czech Republic). The prepared samples were coated with 5 µm of gold prior to SEM analysis.

#### 2.3.2. Tensile Strength Tests

Tensile testing of adhesive compositions was conducted using a Zwick Roell Z150 testing machine (ZwickRoell GmbH & Co. KG, Ulm, Germany), in accordance with PN EN ISO 527-1 (plastics—determination of mechanical properties in static tension) [33]. The crosshead speed during the test was set at 5 mm/min, while the test speed for the tensile modulus was 1 mm/min. An initial tensile force of 30 N was applied.

#### 2.3.3. Compressive Strength Tests

The compressive strength evaluations of the adhesive formulations were also carried out using a Zwick Roell Z150 testing machine, following the guidelines outlined in ISO 604 standard (plastics—determination of properties in compression) [34]. The adopted crosshead travel speed during the test was set at 10 mm/min, while the test speed for the compression modulus was 1 mm/min. An initial test force of 20 N was applied.

#### 2.3.4. Bending Strength Tests

Bending strength tests were conducted using a Zwick Roell Z2.5 testing machine in accordance with the DIN-EN ISO 178 standard (plastics—determination of bending properties) [35]. The testing speed was set at 10 mm/min, with an initial test force of 5 N. The Zwick Roell testing machines used in the tests were operated using TestExpert III software, which facilitated the analysis of the test results obtained.

#### 2.3.5. Sound Absorption

The measurements were conducted using a BSWA SW466 impedance tube (BSWA Technology Co., Ltd., Shanghai, China) configured with two and four microphones, using the transfer-function method according to the ISO 10534-2 standard [36]. The impedance tube offers advantages over other measurement methods due to its compactness, affordability, and rapid result. Compared to the reverberation chamber, the impedance tube requires significantly smaller samples of the tested material. In this setup, a sound-generating speaker is positioned on one end of the impedance tube, while a removable sample holder with a fixed end is located at the other end. Microphones are placed between the speaker and the sample [37,38,39].

The measurements were conducted within a frequency range of 100 Hz to 800 Hz for the first microphone position and from 400 to 2500 Hz for the second position. A total of 18 measurements were performed, divided into three sets of 6 measurements for each frequency band. Subsequently, the measurements were averaged. Each individual measurement lasted 45 s.

This procedure applies to the measurement of both acoustic parameters: the sound absorption coefficient and the reduction index.

**Figure 3:**
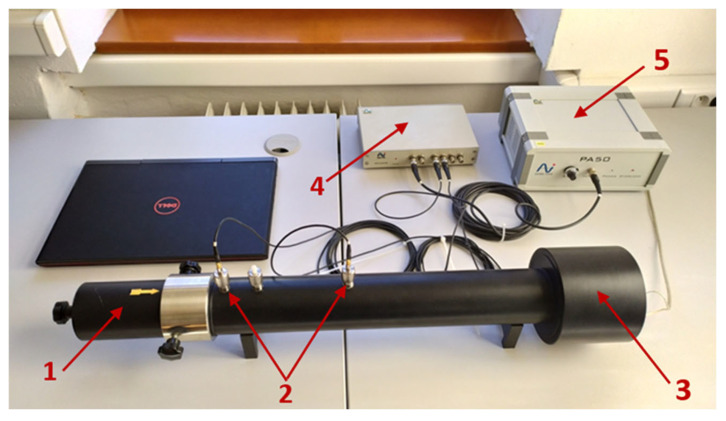
Sound absorption coefficient test station: 1—Sample holder for measuring the sound absorption coefficient; 2—Two microphones for measuring the sound absorption coefficient; 3—Built-in speaker; 4—Four-channel analyzer; 5—Power amplifier.

The sound absorption coefficient (α) is a dimensionless number. An impedance tube in a two-microphone configuration is used to measure the sound absorption factor of selected materials. At one end of the tube, the sample to be examined is placed, while at the other end is a loudspeaker. Figure 3 shows the sound absorption coefficient test station.

The sound absorption coefficient is a measure of a material’s ability to absorb sound energy. It indicates the fraction of the incident sound energy on a material that is absorbed rather than reflected. The value of this coefficient ranges from 0 (total reflection) to 1 (total absorption). The closer the value of the coefficient is to 1, the better the sound absorption of the material. The equation used to determine the sound absorption coefficient is (1):

(1) $$\alpha =\frac{I_{p}-I_{r}}{I_{p}}$$

where

$I_{P}$—incident sound intensity on the surface;

$I_{r}$—reflected sound intensity from the surface.

Thus, α represents the ratio of sound-absorbed energy to the sound-incident energy.

Gumanová et al. define the sound absorption coefficient more extensively in their paper [40].

The reduction index R is a value based on the ratio of the incident sound wave on the front of the sound-absorbing material to the sound waves transmitted back. The reduction index reflects the damping properties of the material, meaning that a higher value indicates greater sound attenuation and better damping properties of the material [41].

**Figure 4:**
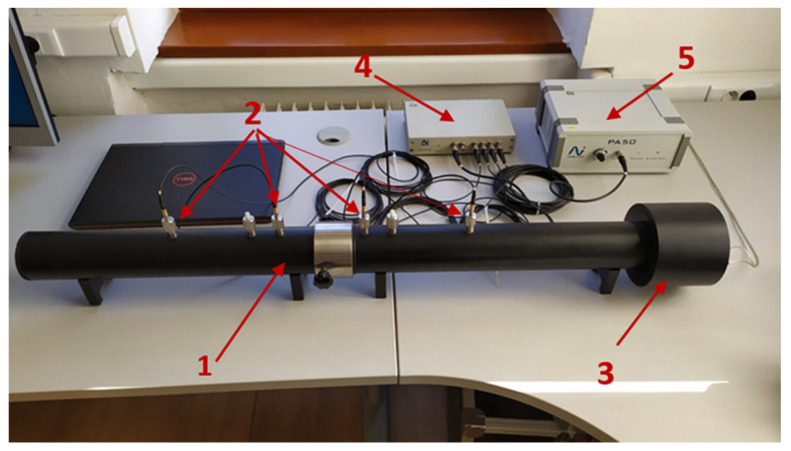
The reduction index *R* test station: 1—Large extension tube for measuring reduction index; 2—Four microphones for measuring reduction index; 3—Built-in speaker; 4—Four-channel analyzer; 5—Power amplifier.

To measure the reduction index of the examined materials, an impedance tube configured with four microphones was utilized. At one end of the tube, a loudspeaker is positioned, and an extension tube is connected. The sample is located in the middle of the tube. Figure 4 shows the reduction index *R* test station.

The sound reduction index *R* is a measure of a material or a building element’s ability to reduce sound transmission, that is, to decrease the sound level after passing through the material. It is typically expressed in decibels (dB) and indicates how much the sound pressure level is reduced by the barrier. The equation used to determine the sound reduction index *R* is (2):

(2) $$R=10\log_{10}\left(\frac{I_{p}}{I_{t}}\right)$$

where

$I_{P}$—incident sound intensity on the barrier;

$I_{r}$—transmitted sound intensity through the barrier. 

In practice, the sound reduction index depends on the frequency of the sound, which is why many standards and regulations use an averaged value (e.g., *R_w_*).

## 3. Results and Discussion

To determine the influence of the filler on specific properties of adhesive compositions, their characteristics were analyzed and juxtaposed with those of unmodified adhesives. The results obtained from testing a series of 10 samples for each material analyzed were subjected to statistical analysis. The average values and the values of statistical deviation were determined, and, in the case of strength properties tests, a test of significant differences was carried out at the assumed significance level *p* = 0.05 to analyze the differences between the groups.

### 3.1. Results of SEM

**Figure 5:**
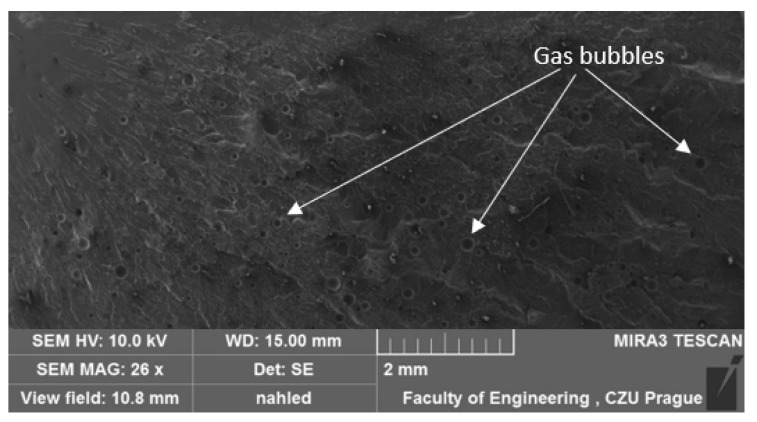
SEM micrographs illustrating the fractured surface of the E5/PAC/100:80 adhesive composition.

**Figure 6:**
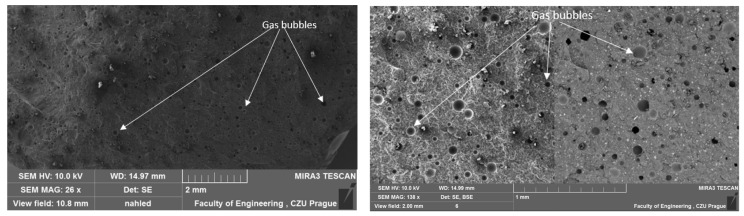
SEM micrographs illustrating the fractured surface of the E5/PAC/ZR2/100:80:1 adhesive composition.

**Figure 7:**
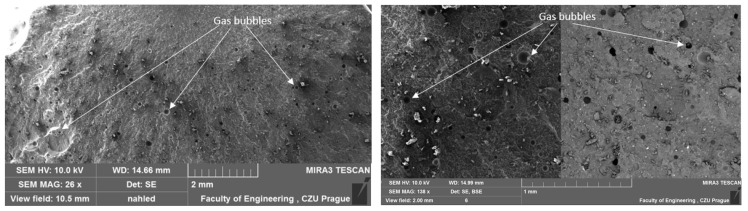
SEM micrographs illustrating the fractured surface of the E5/PAC/CaCO_3_/100:80:5 adhesive composition.

**Figure 8:**
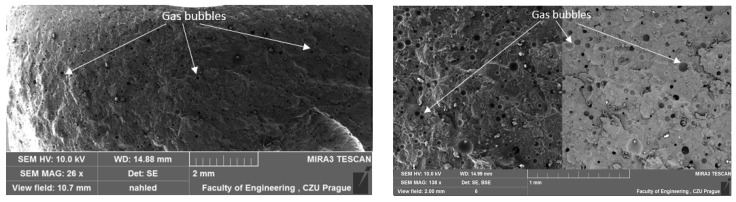
SEM micrographs illustrating the fractured surface of the E5/PAC/CWZ-22/100:80:20 adhesive composition.

A study by Liu Q. et al. [42] indicates that most conventional porous materials typically exhibit good sound absorption performance only at relatively high frequencies. To investigate the structure of the test samples, scanning electron microscopy (SEM) analysis was conducted. The results of this analysis were previously presented in their study [40], focusing on the influence of filler type on the mechanical properties of adhesive compositions and the interfacial interaction between the matrix and the filler. However, in the current discussion, the focus is on the structure and porosity of the adhesive compositions. A comparative depiction of the unmodified reference composition is provided in one of the authors’ papers [43]. The unmodified E5/PAC/100:80 adhesive composition exhibits a homogeneous, solid structure with few visible gas bubbles on the surface. The fracture surface of the reference composition appears smooth and ductile. The structure of the tested adhesive compositions in the cured state is depicted in SEM images in Figure 5, Figure 6, Figure 7 and Figure 8.

The SEM images of all adhesive compositions depicted in Figure 5, Figure 6, Figure 7 and Figure 8 reveal a homogeneous, solid structure. While a few gas bubbles are visible on their surfaces, the structure cannot be considered porous. All compositions exhibit some reproducibility of the fracture. The fractures observed for all compositions are smooth and ductile.

### 3.2. Strength Test Results

**Figure 9:**
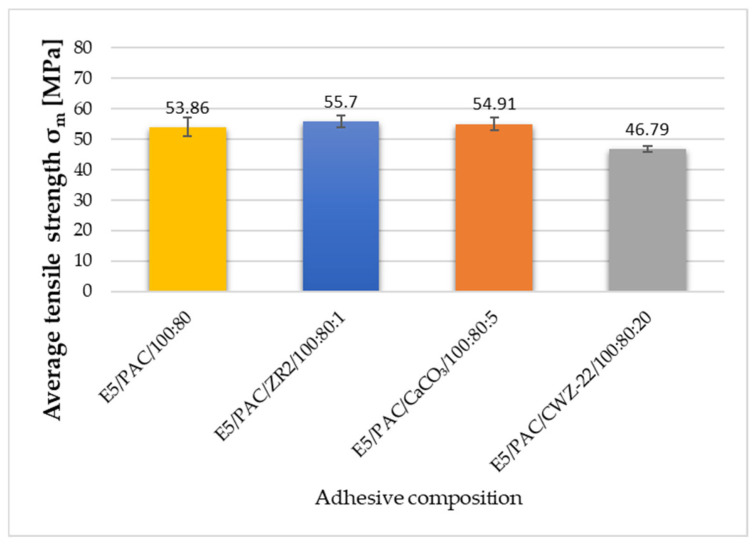
The average tensile strength of the adhesive compositions.

**Figure 10:**
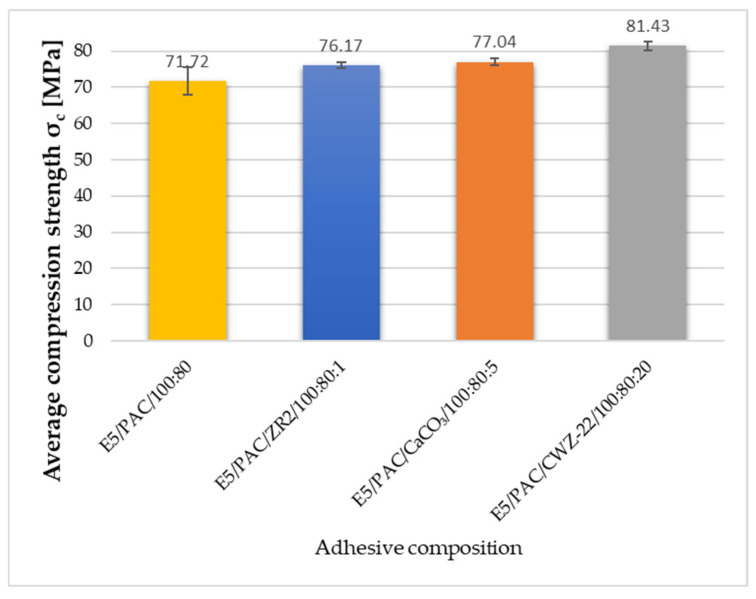
Average compression strength of adhesive compositions.

**Figure 11:**
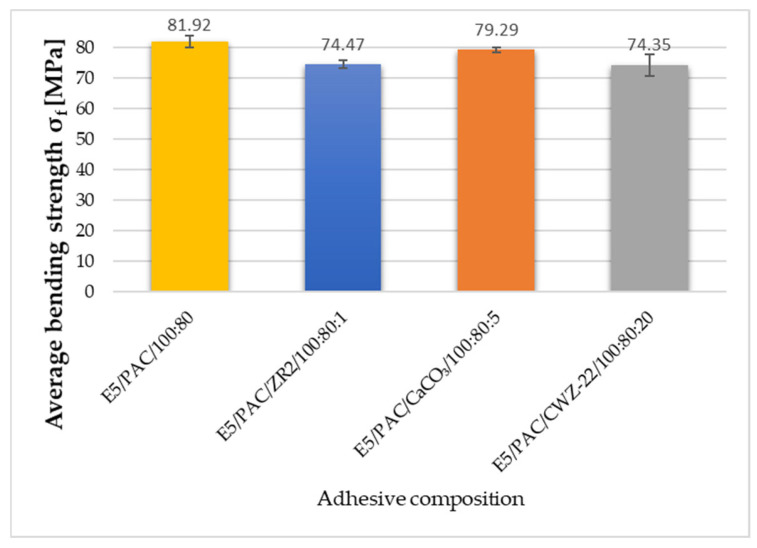
Average bending strength of adhesive compositions.

To discuss the results and to provide a broader overview of the properties of the tested compositions, the results of the strength tests, which were analyzed more extensively in the previous publication, are appended below. The results presented here allow for the reference to be made at the same time to the sound absorption and mechanical properties [43]. The obtained test results are presented in Figure 9, Figure 10 and Figure 11.

Upon analyzing the tensile strength results of the adhesive compositions under study, it is apparent that physical modification had a positive impact on the obtained results for two modified compositions. The highest strength was observed in samples of adhesive compositions with 1% ZR2 filler incorporated. The average tensile strength of the E5/PAC/ZR2/100:80:1 composition was 55.70 MPa. A slightly lower strength, only 1.36%, was observed for compositions enriched with the addition of 5% CaCO_3_ calcium carbonate, resulting in 54.94 MPa. The reference (unmodified) composition exhibited a strength of 53.86 MPa. The lowest strength was recorded for the composition with 20% CWZ-22 filler, measuring 46.79 MPa. However, in this case, the repeatability of the results was at the highest level. The lowest repeatability of results was observed for the reference composition E5/PAC/100:80.

Analyzing the test results obtained in the compression test makes it evident that physical modification had an impact on the compressive strength of the adhesive compositions tested. The highest compressive strength was achieved for compositions containing 20% CWZ-22 activated carbon filler. The average compressive strength of the modified composition E5/PAC/CWZ-22/100:80:20 was 81.43 MPa. A slightly lower strength was observed for the composition containing 5% CaCO_3_ calcium carbonate. The E5/PAC/CaCO_3_/100:80:5 composition had an average compressive strength of 77.04 MPa. A 1.13% lower strength was recorded for the composition modified with 1% ZR2 montmorillonite. The E5/PAC/ZR2/100:80:1 composition displayed a compressive strength of 76.17 MPa. Notably, the results for samples of this composition exhibited the highest repeatability, with a standard deviation of 0.98%. Conversely, the lowest compressive strength was observed for the reference composition E5/PAC/100:80, measuring 71.72 MPa, along with the lowest repeatability of results, indicated by a standard deviation of 5.44%.

Based on the presented results, it is evident that the E5/PAC/100:80 reference composition achieved the highest bending strength. Slightly lower bending strength—by 3.2%—was observed in samples of the adhesive modified with 5% CaCO_3_ calcium carbonate—E5/PAC/CaCO_3_/100:80:5. The average bending strength for this composition was 79.29 MPa. The lowest bending strength in the three-point bending test was recorded for compositions modified with 1% ZR-2 montmorillonite—E5/PAC/ZR2/100:80:1 and 20% CWZ-22 activated carbon—E5/PAC/CWZ-22/100:80:20. These compositions exhibited strengths of 74.47 MPa and 74.35 MPa, respectively. Additionally, the E5/PAC/CWZ-22/100:80:20 composition displayed the widest range of results, with a standard deviation of 4.76%.

**Figure 12:**
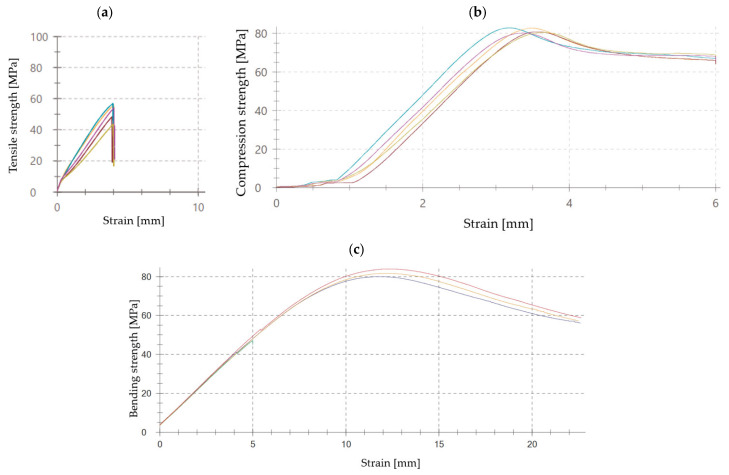
Stress–strain curves: (**a**) Tensile of E5/PAC/100:80 adhesive composition; (**b**) Compression of E5/PAC/CWZ-22/100:80:20; (**c**) Bending of E5/PAC/100:80 adhesive composition.

The strength tests carried out also allowed for the stress–strain curves to be plotted. From there, the stress–strain relationship of the material during the strength test can be analyzed graphically. Figure 12 shows examples of the curves produced during the individual tests.

**Figure 13:**
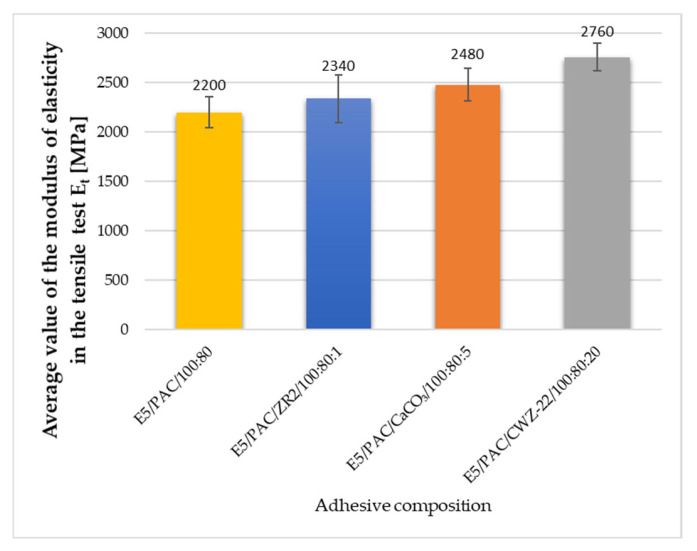
Average value of the modulus of elasticity in the tensile test of adhesive composition.

**Figure 14:**
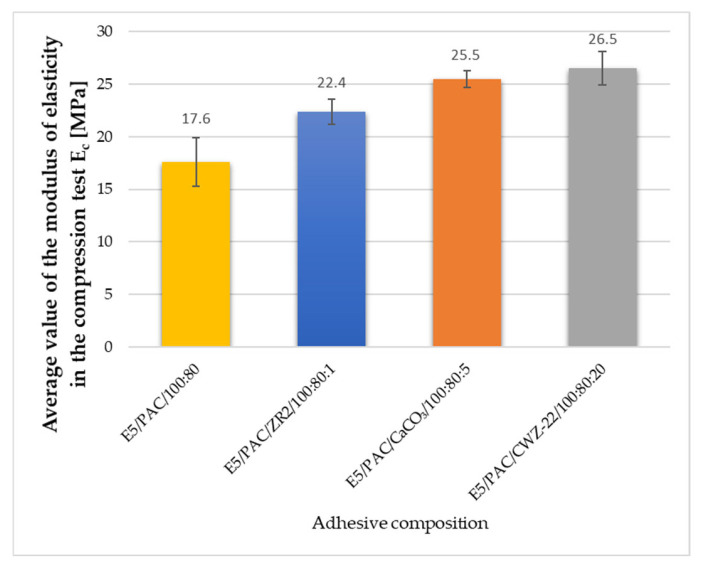
Average value of the modulus of elasticity in the compression test of adhesive composition.

**Figure 15:**
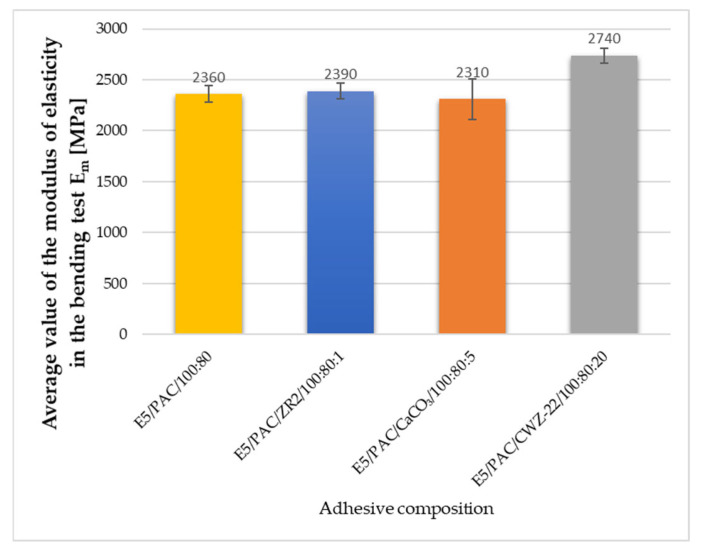
Average value of the modulus of elasticity in the bending test of adhesive composition.

During the strength tests, the modulus of elasticity was also estimated. The average results obtained are shown in Figure 13, Figure 14 and Figure 15.

Elastic modulus, or the ability of a material to resist deformation under load, is a key indicator of its stiffness and stability in various mechanical tests. Based on the conducted studies on epoxy adhesives, the elastic modulus shows significant variations depending on the type of material modification and its properties. The E5/PAC/CaCO_3_/100:80:5 composition exhibited the highest elastic modulus of 2480 MPa, indicating greater stiffness compared to other compositions. The E5/PAC/CWZ-22/100:80:20 composition had the second-highest elastic modulus of 2760 MPa, suggesting high material stiffness. In compression tests, the E5/PAC/CWZ-22/100:80:20 adhesive composition again had the highest elastic modulus of 26.5 MPa, reflecting its high resistance to deformation under compression. The E5/PAC/CaCO_3_/100:80:5 composition had a slightly lower elastic modulus of 25.5 MPa, but it was still higher than most other compositions. In the bending test, the elastic modulus for the E5/PAC/CWZ-22/100:80:20 composition was also the highest at 2740 MPa, suggesting significant resistance to deformation during bending tests. The E5/PAC/CaCO_3_/100:80:5 composition had an elastic modulus of 2310 MPa, indicating considerable stiffness but somewhat lower compared to the CWZ-22 modified composition.

The modulus of elasticity is crucial in engineering design, particularly in analyzing how materials behave under load. A higher modulus of elasticity means that the material is more rigid and less susceptible to deformation under stress. A high elastic modulus does not necessarily mean high tensile strength, as can be perfectly observed in the example of the E5/PAC/CWZ-22/100:80:20 adhesive composition. A similar relationship can also be observed by analyzing the modulus of elasticity determined in the compression test.

### 3.3. Sound Absorption Test Results

**Figure 16:**
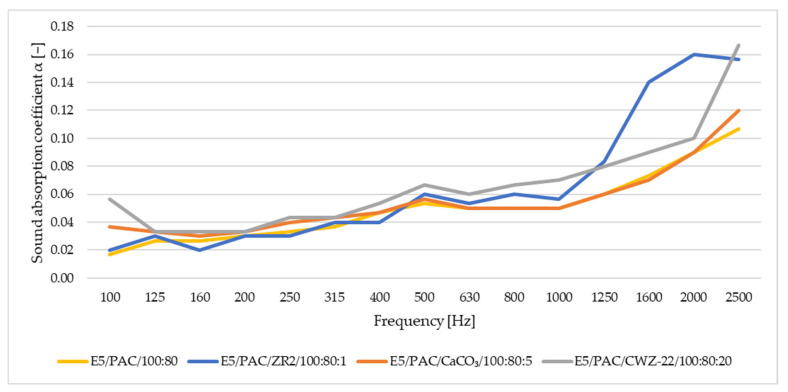
Sound absorption coefficient for samples with a thickness of 10 mm.

**Figure 17:**
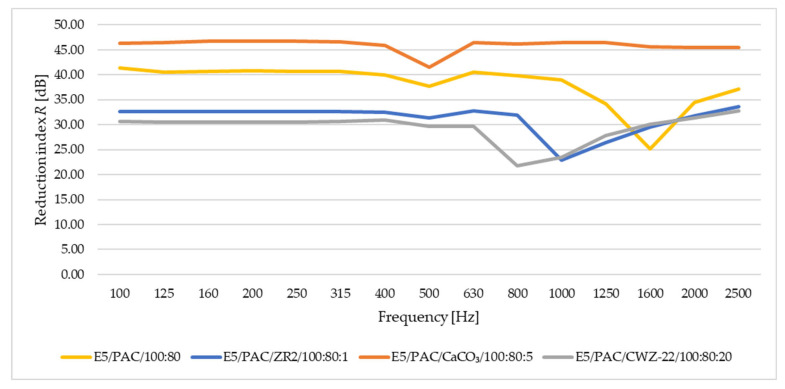
Reduction index *R* for samples with a thickness of 10 mm.

**Figure 18:**
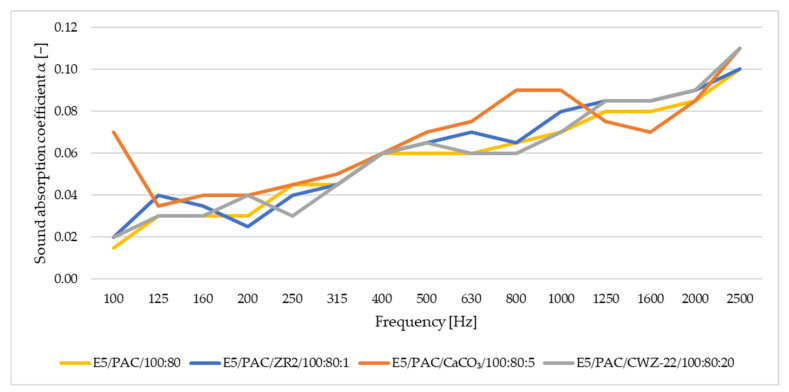
Sound absorption coefficient for samples with a thickness of 60 mm.

**Figure 19:**
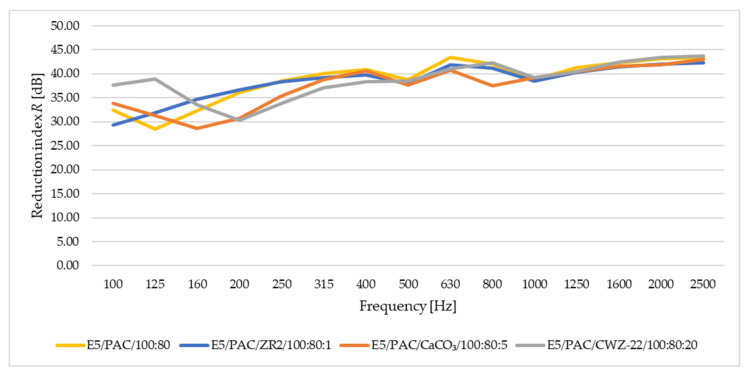
Reduction index R for samples with a thickness of 60 mm.

In this study of sound adsorption, both the sound absorption coefficient and the reduction index were analyzed. The results obtained are depicted in Figure 16, Figure 17, Figure 18 and Figure 19. Figure 16 shows the results of sound absorption coefficient analysis for 10 mm thick samples.

From the results for samples with a thickness of 10 mm, it is apparent that the values of the sound absorption coefficient increase with increasing frequency. However, despite this trend, the materials exhibit low values of sound absorption, reaching a maximum of around 0.18.

Figure 17 provides a summary of the results for the reduction index *R* for samples with a thickness of 10 mm.

From the perspective of the reduction index *R*, it is evident that these samples yield better results than absorption, meaning that over the entire frequency range set during the test, the results are more stable. The highest values are achieved by the E5/PAC/CaCO_3_/100:80:5 material across the entire frequency range.

Figure 18 shows the results of the sound absorption coefficient test for samples with a thickness of 60 mm.

For the thicker 60 mm samples, a similar trend is observed for the thinner 10 mm samples. The values of the sound absorption coefficient increase with frequency, but overall, they remain low for all materials. Minimal differences are observed between individual materials.

Figure 19 shows the reduction index *R* for samples with a thickness of 60 mm.

With the reduction index *R*, only minimal differences are observed between the individual materials. However, in terms of reduction index *R*, the materials achieve better values than sound absorption, which also means that over the entire frequency range set during the test, the results are more stable.

When analyzing the effect of sample thickness on the sound absorption coefficient α and sound reduction index *R*, the sound absorption capacity of the materials analyzed is limited due to their stiffness and low porosity. Perhaps other factors, such as surface area and texture, rather than thickness alone, would have a greater influence.

## 4. Conclusions

The experimental study presented in this paper investigated epoxy adhesive compositions subjected to physical modification by introducing modifying additives in the form of fillers. The focus was on verifying the relationship between the mechanical properties of modified epoxy adhesives, their structure, and sound absorption performance. Based on the obtained results, several important conclusions can be formulated:

- Physical modification of epoxy adhesive compositions does not always yield positive results;
- In the cases of analyzed adhesive compositions, the changes in strength parameters were generally insignificant, with significant differences observed at the assumed significance level *p* = 0.05 only in the following case:
- Tensile strength: A significant decrease for the composition E5/PAC/CWZ-22/100:80:20 compared to the reference composition;
- Compressive strength: A significant increase in strength for all modified compositions;
- Bending strength: A significant decrease for the compositions E5/PAC/ZR2/100:80:1 and E5/PAC/CWZ-22/100:80:20 compared to the reference composition;
- Regarding the sound absorption test, it can be observed that all tested materials show low values of the sound absorption coefficient; therefore, low sound absorption in both thicknesses was investigated (10 mm and 60 mm). Only minimal differences are noticeable between the different materials in terms of sound absorption;
- For the reduction index, better results are observed than for sound absorption for all materials. Only minimal differences between the individual materials are noticeable, but stable values are seen across the frequency band;
- Analyzing the SEM images of the tested compositions, it is evident that gas bubbles are visible on the surfaces, but the structure cannot be concluded to be porous in nature. In the case of the modified compositions, more gas bubbles are present compared to the reference composition, which may be due to the double-mixing step;
- Different fillers affect mechanical properties such as tensile, compressive, and bending strengths, with some enhancing strength and others improving specific properties;
- The adhesives generally exhibit low sound absorption, which can be attributed to their non-porous, solid structure;
- The reduction index *R* values are higher, indicating better sound reduction performance compared to sound absorption;
- The non-porous structure of the adhesives limits their sound absorption capabilities, while their homogeneous structure contributes to the stability of their mechanical properties;
- Adding different fillers into epoxy adhesive compositions significantly affects the elastic modulus. For instance, the CWZ-22 filler shows a markedly higher elastic modulus compared to other fillers, indicating its ability to increase material stiffness;
- A higher elastic modulus does not always correlate with higher tensile, compressive, or bending strength. For example, the E5/PAC/CWZ-22/100:80:20 composition, despite having the highest elastic modulus, did not always achieve the highest strength values across different tests.

In conclusion, the analyzed physical modification of the adhesive compositions did not contribute to a change in sound absorption properties. However, it should be noted that this statement is correct for the materials and preparation technology of the adhesive compositions used in the presented studies. As pointed out in the introduction, porous materials are much better at sound attenuation than materials with a solid structure because the porous structure allows for the sound waves to be absorbed by dissipating their energy, thus attenuating sound rather than reflecting it. The relationship between the mechanical properties, structure, and sound absorption of the tested epoxy adhesives highlights that while physical modifications can enhance strength, they do not significantly improve sound absorption due to the inherent lack of porosity. The high modulus of elasticity and strength of certain compositions reflect their suitability for structural applications, whereas their sound absorption capabilities are limited by their non-porous nature.

Therefore, future research will attempt to modify the preparation of the compositions, change the range of fillers used, and alter the curing agents to increase the porosity of the material while maintaining high strength parameters of the adhesive compositions.

## Figures and Tables

**Table 1:** The physical and chemical attributes of Epidian 5 epoxy resin [22,23,24].

Properties	Epidian 5 Epoxy Resin
Epoxy number	0.48–0.52 mol/100 g
pH value	approx. 7
Viscosity at 25 °C	20,000–30,000 mPa·s
Density at 20 °C	1.16 g/cm^3^
Flash point	266 °C
Auto-ignition temperature	490 °C
Melting point	30–50 °C
Initial boiling point	not indicated—degradation

**Table 2:** Functional properties of the PAC curing agent [23].

Properties	PAC Curing Agent
Viscosity at 25 °C	10,000–25,000 mPa·s
Density at 20 °C	1.10–1.20 g/cm^3^
Amine number	290–360 mg KOH/g
Gelling time (example for composition with Epidian 5 at 20 °C)	180 min

**Table 3:** Properties of montmorillonite ZR2 NanoBent.

Parameter	ZR2 NanoBent
Form	cream-colored lamellar powder
Water content	≤3.0% wag.
650 °C roasting loss	25–30% wag.
Swelling in xylene	>20% obj.
Vapour absorption of white spirit 48 h	>20% wag.
Bulk density	<0.5 g/cm^3^
Ion exchange capacity CEC of bentonite raw material	min. 80 mmol/100 g dry bentonite raw material

**Table 4:** Basic physical and chemical properties of CaCO_3_ used in this study.

Properties	CaCO_3_ Calcium Carbonate
Form	light-grey solid in various sizes: lumps or fine powder
pH value	9.2 (at 25 °C)
Temperature melting point	>450 °C (degradation temperature −825 °C)
Flammability	non-flammable
Limits explosiveness	non-explosive (free of any chemical structures associated with explosive properties)
Relative density	2.711 g/cm^3^ (at 20°)
Solubility in water	14 mg/dm^3^ (at 25 °C)
Viscosity	not applicable (solid with melting point > 450 °C)
Explosive properties	non-explosive (free of any chemical structures associated with explosive properties)
Oxidizing properties	has no oxidizing properties (based on the chemical structure, the substance does not contain excess oxygen or any structural group tending to react exothermically with the combustible material)
Degradation temperature	825 °C
Bulk weight	(0.7–1.4) 10^6^ g/m^3^ (at 20 °C)
Electrostatic properties	the substance does not generate electrostatic charges

**Table 5:** Basic properties of CWZ-22 activated carbon.

Properties	CWZ-22 Activated Carbon
Form	solid, dusty black color
pH value	about 6 (50 g/L H_2_O as a suspension, 20 °C)
Melting point	no data, sublimation about 3700 °C
Explosive limits	lack of data
Relative density	approximately 2 g/cm^3^
Solubility in water	in water: insoluble, in organic solvents: no data
Bulk weight	about 400 · 10^3^ g/m^3^

**Table 6:** Labeling of the adhesive formulations utilized in these experiments.

Epoxy Resin	Curing Agent	Filler	Identification
Epidian 5 (100 g)	PAC (80 g)	-	E5/PAC/100:80
		ZR2 NanoBent (1 g)	E5/PAC/ZR2/100:80:1
		CaCO_3_ calcium carbonate (5 g)	E5/PAC/CaCO_3_/100:80:5
		CWZ-22 activated carbon (20 g)	E5/PAC/CWZ-22/100:80:20

**Table 7:** Shape and dimensions of the samples to be tested.

Type of Testing	Shape and Dimensions of Samples, Dimensions in Millimetres
Tensile strength test The dump-bell type 1B sample [33]	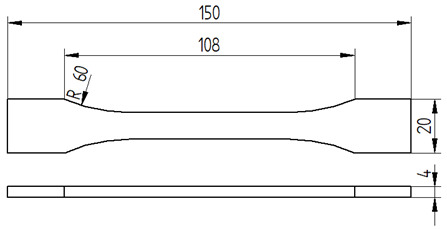
Compression strength test Cylindrical sample [34]	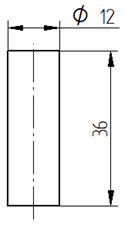
Bending strength test The beam sample, in accordance with ISO 178:2003 standard [35]	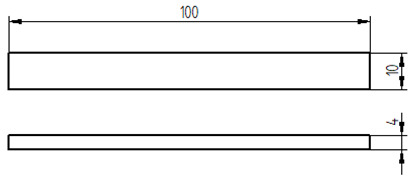
Sound Absorption, in accordance with ISO 10534-2 standard [36]	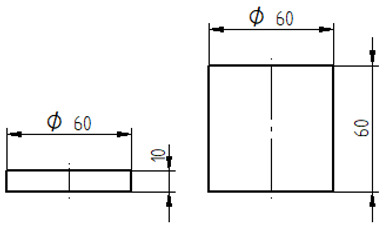
Scanning electron microscopy (SEM)	Gold sputtering of 100 × 10 × 4 mm beam samples using Quorum Q150R ES-Spreading Deposition Rate sputtering machine (Quorum, Laughton, UK)

## Data Availability

The raw/processed data required to reproduce these findings cannot be shared at this time due to technical or time limitations. Data can be made available upon individual request.
